# Construction of a Viscoelastic Model of Human Cancellous Bone in Alveolar Bone Based on Bone Mineral Density Distribution

**DOI:** 10.3390/ma16237427

**Published:** 2023-11-29

**Authors:** Bin Wu, Le Yuan, Mao Liu, Miaoning Tang, Di Jiang, Yang Yi, Songyun Ma, Bin Yan, Yi Lu

**Affiliations:** 1 College of Mechanical and Electronic Engineering, Nanjing Forestry University, Nanjing 210037, China; wubin@njfu.edu.cn (B.W.); yl230706@163.com (L.Y.); jiangdijd@163.com (D.J.); yiyang_njfu@163.com (Y.Y.); 2 Department of Orthodontics, Affiliated Hospital of Stomatology, Nanjing Medical University, Nanjing 210029, China; maoliu@stu.njmu.edu.cn (M.L.); 18851729106@163.com (M.T.); 3 Jiangsu Province Key Laboratory of Oral Diseases, Nanjing 210029, China; 4 Jiangsu Province Engineering Research Center of Stomatological Translational Medicine, Nanjing 210029, China; 5 Institute of General Mechanics, RWTH-Aachen University, 52062 Aachen, Germany; ma@iam.rwth-aachen.de

**Keywords:** human cancellous bone in alveolar bone, bone mineral density, dynamic mechanical analysis, dynamic viscoelasticity, constitutive model

## Abstract

Orthodontic treatment was accompanied by the remodeling of cancellous bone in alveolar bone (CBAB), which manifested as the increase or decrease in bone mineral density (BMD). BMD is closely related to the mechanical properties of the alveolar bone. Therefore, the aim of this study was to quantify the effect of BMD on its viscoelastic behavior and to assess orthodontic forces at different BMDs. A total of nine CBAB samples were cut from the cervical, middle, and apical regions of the right mandible between canine, premolars, and molars. After scanning with micro-computed tomography (micro-CT). The BMD of samples was measured and dynamic mechanical analysis (DMA) was performed. Based on the fourth-order generalized Maxwell model, a viscoelastic constitutive model characterizing the BMD variation was constructed. The BMD exhibited variations within different regions of the CBAB. The storage modulus is positively correlated with BMD, and the loss modulus is negatively correlated with BMD.

## 1. Introduction

Alveolar bone is divided into three parts: intrinsic alveolar bone, cancellous bone, and cortical bone [1]. Alveolar bone changes actively during orthodontic treatment, which is bone remodeling. Cancellous bone in alveolar bone (CBAB) is mainly composed of a complex three-dimensional reticular arrangement of bone trabeculae, which are considered viscoelastic materials [2,3,4], and their mechanical properties are influenced by the load and bone mineral density (BMD) [5]. The remodeling of the alveolar bone is the reason for the change in BMD. In general, the trabecular bone will have significant morphological differences due to different stress conditions or due to different parts [6]. In addition, with the change in trabecular bone morphology, the mechanical response is different [7]. Therefore, in orthodontic clinical work, it is necessary to further clarify the effect of BMD on the viscoelastic mechanical properties of CBAB, so as to facilitate the rational reconstruction of alveolar bone and the healthy movement of orthodontic teeth.

Some scholars have studied the BMD of teeth and its effect on orthodontic treatment. The BMD in the anterior region of the human jaw is larger than that in the posterior region [8]. The value of BMD also affects the level of tooth movement during orthodontic treatment. It shows that in all types of motion simulations, higher tooth movement was obtained at lower BMD [9]. Through the experiment and monitoring of rats, it was found that BMD changed dynamically over different orthodontic time periods [10]. Thom Bitter et al. performed compression tests on the bone trabeculae of the human tibia. Three basic material parameters were obtained as a function of BMD to develop an isotropic crushable foam (CF) model. Dynamic mechanical analysis (DMA) describes the properties of materials through the state of molecular motion [11]. This has been widely used to characterize the viscoelastic properties of composites and biomaterials [12]. It can measure viscoelastic parameters, including storage modulus (*E*′) and loss modulus (*E*″), which reflect the elastic and viscous properties of materials, respectively [13,14]. Refs. [15,16] investigated the effect of BMD on the viscoelasticity of articular cartilage in bovine femoral heads via DMA. However, most of the early studies focused on animals [17] or other parts of human cancellous bone [18]. There were fewer studies on the microstructure of human CBAB and its mechanical properties. The orthodontic process was accompanied by static occlusion and dynamic mastication. The dynamic force plays a pivotal role in orthodontic treatment [19], but most studies on CBAB are static tests [20], and the response of human CBAB after dynamic loading has not been studied. Moreover, most of the previous studies on alveolar bone have simplified its structural model [21], which cannot reflect its non-homogeneous characteristics.

In this paper, a human CBAB constitutive model was constructed based on the distribution of BMD. The samples used were taken from the mandibles of fresh cadavers, and the BMD of different regions of CBAB were measured based on micro-CT imaging, and the viscoelastic properties of each sample were tested separately. Moreover, experimental data were used to fit and prove the constitutive model. This study provides a reference for the further study of the microporous medium model and has guiding significance for applying appropriate orthodontic force during orthodontic treatment.

## 2. Material and Methods

### 2.1. Sample Preparation and BMD Measurement

**Figure 1:**
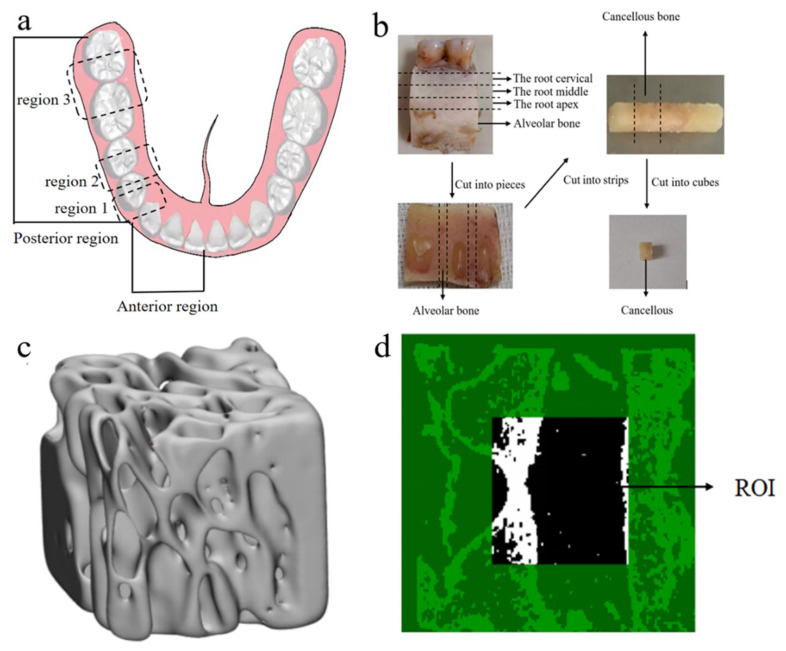
CBAB samples. (**a**) Schematic diagram of the tooth region. (**b**) Sample preparation process (**c**) CBAB reconstruction image under micro-CT. (**d**) Selected region of interest (ROI).

This study was reviewed and approved (No. (2020)234) by the Institutional Review Board (IRB) of Nanjing Medical University. All samples in this experiment were stored in a refrigerator at −20 °C and thawed at room temperature before use. Initially, mandibular fragments from one human cadaver were used to create samples, and the soft tissues on the surface of the mandibular alveolar bone were carefully removed. The low-speed cutters (Isomet, Buehler, Lake Bluff, IL, USA) were used to obtain 2 mm-thick sections of the cervical, apical, and mid-root regions of the mandible at the sites of canine (region 1), premolars (region 2), and molars (region 3) and were cut perpendicular to the long axis of the teeth, as shown in Figure 1a.

Then, the slices were cut into long strips, and finally, cut into cubes. A total of 9 cube samples (2 × 2 × 2 mm) were prepared. They were stored in a test tube containing a saline solution in the refrigerator. The preparation process of CBAB samples is shown in Figure 1b.

### 2.2. Mechanical Experiments

All the cube samples were scanned using micro-computed tomography (micro-CT) (ScancoMedical, Bassersdorf, Switzerland) at a resolution of 15.6 μm. The scanning parameters used were source voltage of 55 kV and a current of 72 μA. Each projection was rotated by 0.4°, with an average of 8 frames per projection and an exposure time of 120 s. CT images revealed that the CBAB is a complex porous structure composed of trabecular bone (Figure 1d).

Skyscan software was used to reconstruct the image, CTAn software was used to select a 20-layer, 50 × 50-pixel square as the region of interest, and the BMD of the sample was measured based on the gray value of the image, as shown in Figure 1c,d. BMD was measured five times by the same person, and the average value was taken as the BMD value. Due to the preciousness of human alveolar cancellous bone, this study did not carry out biological repetition experiments. The cancellous bone in the alveolar of the same person was tested. The BMD of each sample was measured multiple times by the same person, and the average value was used as the BMD.

**Figure 2:**
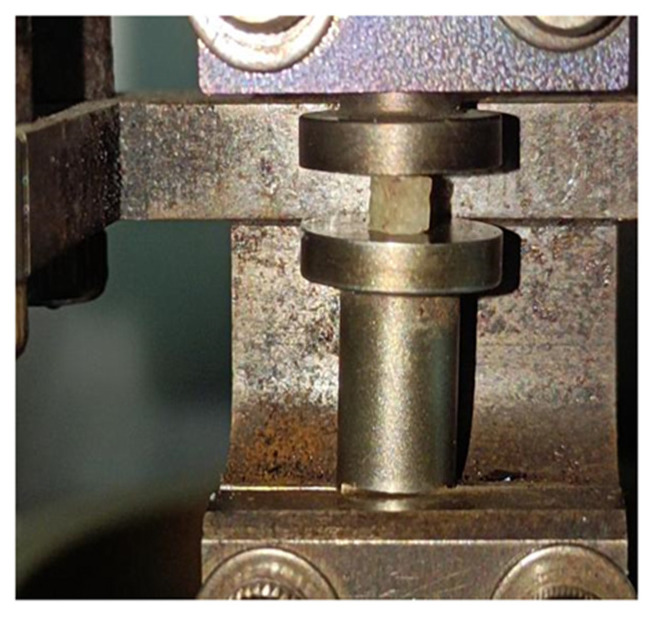
Dynamic mechanical analyzer.

Before the test, the samples were thawed at room temperature, then the DMA procedure was applied to the cube samples using a Pyris Diamond Dynamic Mechanical Analyzer (Perkin Elmer, Waltham, MA, USA). CBAB samples were loaded along the vertical direction of the fixture, a preload of 0.5 mN was applied to ensure sufficient contact between the samples and the fixture, as shown in Figure 2. The tests were performed in compression mode with a constant temperature of 25 °C, and the amplitude of the dynamic compression force was 5 N. Each sample was tested at four frequencies (0.5, 1, 2, and 5 Hz) for 20 min each, and the dynamic viscoelastic modulus of CBAB samples with different BMD at different frequencies was obtained.

## 3. Results

### 3.1. Analysis of Results of BMD Measurement

The bone mineral density measured via micro-CT was shown in Table 1, and there were obvious differences in the trabeculae of CBAB at different regions, with some regions displaying denser trabeculae and some regions displaying sparser trabeculae. When BMD was low, there was a noticeable decrease in the number of trabecular bones, while higher BMD levels correspond to a denser trabecular bone structure. The number of bone trabeculae corresponding to each bone mineral density is shown in Table 2.

On the other hand, the BMD of CBAB varied across different tooth regions. It can be seen from Table 1 that the BMD of the anterior teeth region was larger than that of the posterior teeth region. Furthermore, the BMD also exhibited variations within different regions of the same tooth region, with the root cervical region showing significantly higher values compared to the root and apical regions.

### 3.2. The Influence of BMD on Viscoelastic Modulus

**Figure 3:**
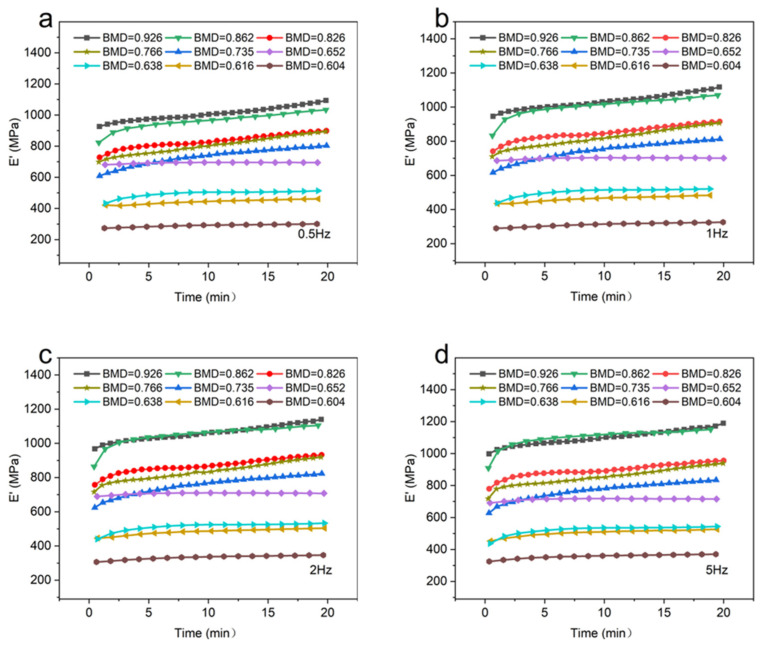
The storage modulus of different bone mineral density samples. (**a**): 0.5 Hz; (**b**): 1 Hz; (**c**): 2 Hz; (**d**): 5 Hz.

The storage modulus (*E*′) of CBAB with different BMD at room temperature (25 °C) was shown in Figure 3.

It can be seen that at the same temperature and frequency, the BMD of CBAB increased from 0.604 g/cm^3^ to 0.926 g/cm^3^ and the storage modulus also increased gradually in general. For samples with lower BMD (BMD = 0.652, BMD = 0.638, BMD = 0.616, BMD = 0.604), the storage modulus demonstrated a relatively gradual and linear change over time. At intermediate BMD levels (BMD = 0.826, BMD = 0.766, BMD = 0.735), the storage modulus exhibited an upward trend, as evidenced by an increasing slope in the curve. In the case of CBAB samples with higher BMD (BMD = 0.926, BMD = 0.862), it was evident that the storage modulus initially underwent a rapid increase, followed by a steady rise. With the increase in frequency, the duration of the rising stage was longer. From 1 Hz to 5 Hz, the average storage modulus of CBAB samples with lower BMD increased by 12%, and the storage modulus of CBAB samples with higher BMD increased by 23%.

**Figure 4:**
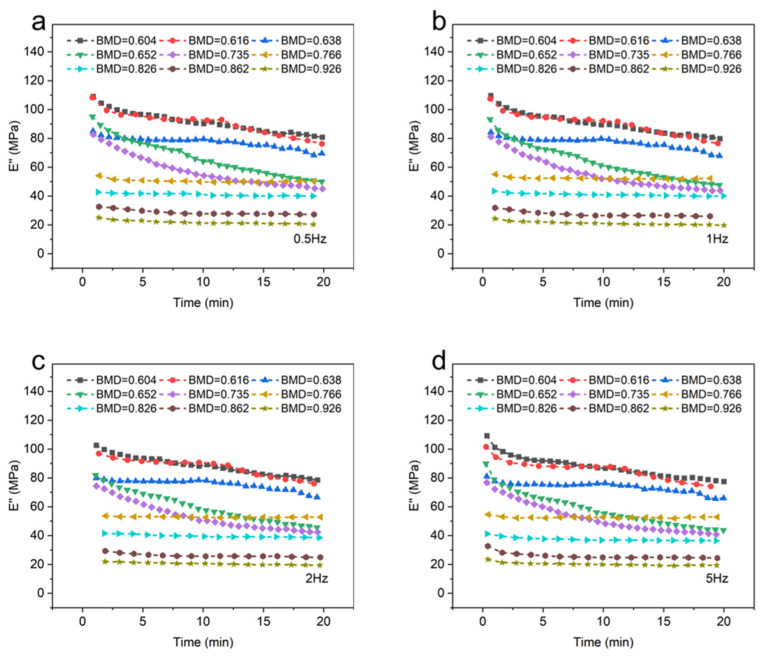
The loss modulus of different bone mineral density samples. (**a**): 0.5 Hz; (**b**): 1 Hz; (**c**): 2 Hz; (**d**): 5 Hz.

Figure 4 depicts the loss modulus (*E*″) of different BMD samples. When the temperature and frequency were consistent, a negative correlation was observed between the BMD and the loss modulus of CBAB.

As shown in Figure 4a–d, the viscosity of CBAB decreased after being subjected to oscillating force. An increase in BMD indicated an increase in the number of bone trabeculae, implying the decrease in viscous substances in alveolar bone with the same volume, such as bone marrow, which led to the decrease in loss modulus. At the same time, when BMD was large (BMD = 0.926, BMD = 0.862, BMD = 0.826, BMD = 0.766), the loss modulus decreased by only 12%. Notably, when the BMD was below 0.766, a significant change in the loss modulus was observed. Specifically, for smaller BMD values (BMD = 0.652, BMD = 0.616, BMD = 0.604), the loss modulus was noticeably reduced by 31%.

Furthermore, Figure 3 and Figure 4 demonstrate the existence of BMD thresholds for both the storage modulus and loss modulus. Beyond these thresholds, the variations in the modulus values became significantly pronounced, indicating a more substantial impact on the material’s behavior.

### 3.3. The Impact of Frequency on the Viscoelastic Modulus

**Figure 5:**
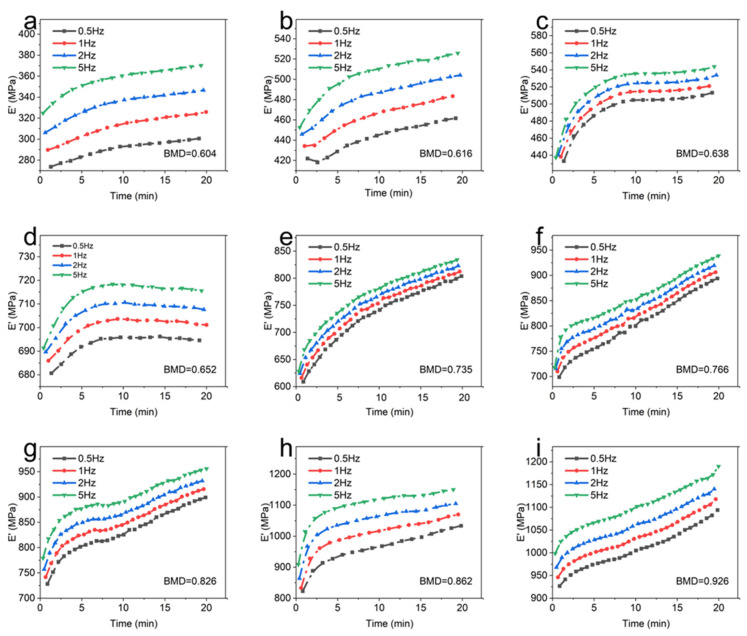
The storage modulus of each sample at different frequencies. (**a**): BMD = 0.604; (**b**): BMD = 0.616; (**c**): BMD = 0.638; (**d**): BMD = 0.652; (**e**): BMD = 0.735; (**f**): BMD = 0.766; (**g**): BMD = 0.826; (**h**): BMD = 0.862; (**i**): BMD = 0.926.

Figure 5 compares the influences of frequency on the viscoelastic modulus of CBAB.

It demonstrates that the storage modulus of the sample exhibits an upward trend as the frequency increases from 0.5 Hz to 5 Hz at room temperature. In addition, the effect of frequency on the storage modulus of CBAB varied with BMD. For example, the maximum variations of the storage modulus of the CBAB sample with the BMD of 0.862 g/cm^3^ at 0.5 Hz to 5 Hz were 26%, 28%, 28%, and 27%, respectively, while the BMDs of 0.604 g/cm^3^ were 9%, 12%, 13% and 14%, respectively. Notably, the average storage modulus of the samples with smaller BMDs varied little at four frequencies. For CBAB samples with larger BMD, its storage modulus was more susceptible to frequency, but the overall change range of the storage modulus remained relatively small.

**Figure 6:**
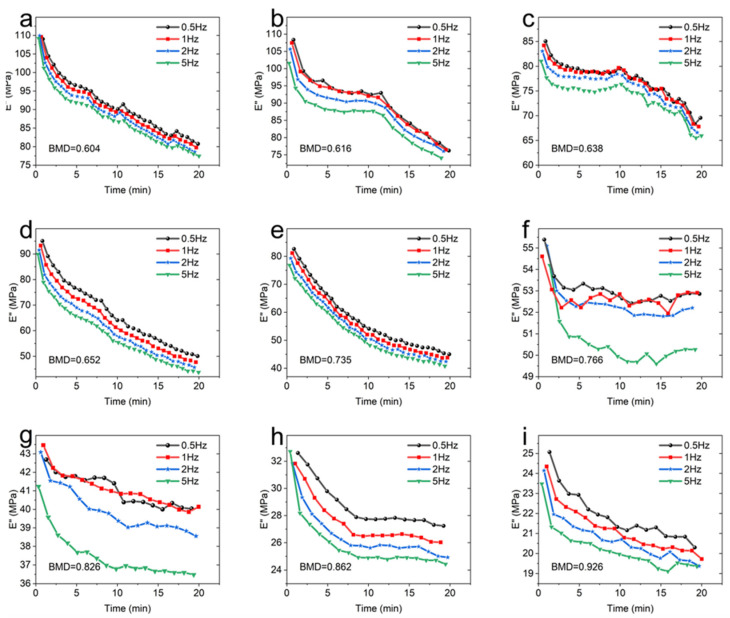
The loss modulus of each sample at different frequencies. (**a**): BMD = 0.604; (**b**): BMD = 0.616; (**c**): BMD = 0.638; (**d**): BMD = 0.652; (**e**): BMD = 0.735; (**f**): BMD = 0.766; (**g**): BMD = 0.826; (**h**): BMD = 0.862; (**i**): BMD = 0.926.

Figure 6 describes the influence of frequency on the loss modulus. It can be seen from Figure 6 that the loss modulus decreased with the increase in frequency.

When the BMD was 0.735 and below, the frequency had little effect on the loss modulus of CBAB. Similarly, the effect of frequency on CBAB loss modulus also varied with BMD. The loss modulus of samples with small BMD was more susceptible to frequency. For example, the maximum variations of the loss modulus of the CBAB sample with BMD of 0.604 g/cm^3^ at 0.5 to 5 Hz were 26%, 27%, 28% and 29%, respectively, while the BMDs of 0.926 g/cm^3^ were 19%, 19%, 20% and 18%, respectively.

Under identical temperature conditions, the trend of the viscoelastic modulus of CBAB samples remained consistent with varying frequencies. The variation in the external force period from 0.5 Hz to 5 Hz was minimal and had little impact on the movement of the molecular chains within the CBAB. Consequently, the change in the viscoelastic modulus was also negligible within this frequency range. For example, when the frequency was increased by a factor of 10 (Figure 5 and Figure 6), The average change rates of storage modulus and loss modulus were only about 25% and 28%, respectively, whereas the average change rates of storage modulus and loss modulus were about 281% and 82%, respectively, when the BMD increased by 53%. In summary, the effect of frequency on the overall change of viscoelastic modulus was much smaller than that of BMD, and the effect of frequency was not considered when establishing the viscoelastic model.

### 3.4. Viscoelastic Constitutive Model Based on BMD Distribution

The generalized Maxwell model can accurately describe the stress–strain relationship for viscoelastic materials; it consists of an n-cell Maxwell model and a parallel spring.

The relaxation modulus expression of the generalized Maxwell model *E*(*t*) is:

(1) $$E\left(t\right)=E_{\infty }+\sum_{i=1}^{n}E_{i}\cdot {\mathrm{e}}^{-\frac{t}{\tau_{i}}}$$

where $E_{\infty }$ is the equilibrium relaxation modulus, which represents the storage modulus when the angular frequency is 0.

By performing Fourier transform and inverse transform on Equation (1), it can be expressed as:

(2) $$E^{\prime }=E_{\infty }+\sum_{i=1}^{n}E_{i}\frac{\omega^{2}\tau_{i}^{2}}{{1+\omega }^{2}\tau_{i}^{2}}$$

(3) $$E^{\prime \prime }=\sum_{i=1}^{n}E_{i}\frac{\omega \tau_{i}^{2}}{{1+\omega }^{2}\tau_{i}^{2}}$$

where *E*′ is the storage modulus, *E*″ is the loss modulus, and ω is the frequency.

Before fitting the model parameters, Equations (2) and (3) need to be converted into Prony series expressions:

(4) $$E^{\prime }=E_{\infty }+E_{0}\sum_{i=1}^{n}\frac{g_{i}\tau_{\mathrm{i}}^{2}\omega^{2}}{1+\tau_{i}^{2}\omega^{2}}$$

(5) $$E^{\prime \prime }=E_{0}\sum_{i=1}^{n}\frac{g_{i}\tau_{i}\omega }{1+\tau_{i}^{2}\omega^{2}}$$

where $E_{0}=\frac{E_{\infty }}{1-\sum_{i=1}^{n}g_{i}}$ is the instantaneous modulus, $g_{i}=\frac{E_{i}}{E_{0}}$ is the dimensionless modulus parameter, $\tau_{i}=\frac{\eta_{i}}{E_{i}}$ is the relaxation time, ω is the frequency, and n is the model order.

The fourth-order model (*n* = 4) has the advantages of high efficiency, low error, and convenient parameter acquisition [22]. Therefore, in this paper, we fitted the viscoelastic parameters based on the fourth-order generalized Maxwell model and experimental response and established a viscoelastic constitutive model that characterizes changes of BMD. 

A function of the model parameters was obtained by minimizing the sum of squares of the errors D between the experimental data and the estimated data using nonlinear least squares, using the expression

(6) $$D=\sum_{i=1}^{n}\left[{\left(\bar{E^{\prime }}-\bar{E^{\prime }}\right)}^{2}+{\left(E^{\prime \prime }-\bar{E^{\prime \prime }}\right)}^{2}\right]$$

where $\bar{E^{\prime }}$ is the fitted value of storage modulus, $\bar{E^{\prime }}$ is the experimental value of storage modulus, $E^{\prime \prime }$ is the fitted value of loss modulus, and $\bar{E^{\prime \prime }}$ is the experimental value of loss modulus.

At the same time, in order to narrow the search range of the unknown quantity, improve the computational efficiency, and ensure the reasonableness of the obtained parameters, the parameter relaxation time and parameters were constrained as follows:

(7) $$\tau_{i}>0$$

(8) 0<gi<1

**Figure 7:**
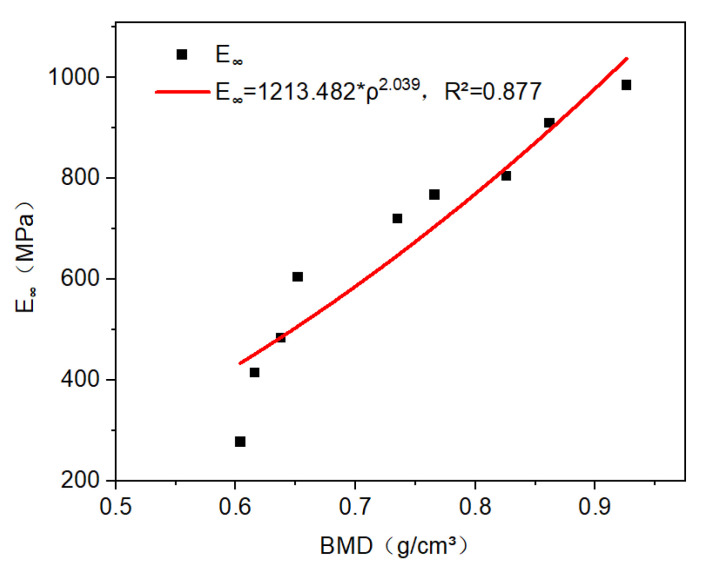
The relationship between equilibrium modulus and BMD is power–law.

For CBAB, its viscoelastic modulus was greatly affected by BMD. The equilibrium modulus ($E_{\infty }$) for all CBAB ranged from 260 to 985, which varied with BMD (ρ). It can be described by an exponential function, such as ${E_{\infty }=1213.482\times \rho^{2.039}}_{}$(r^2^ = 0.877). With the increase in BMD, the equilibrium modulus increased, as shown in Figure 7. This relationship was consistent with the modulus–BMD relationship of bone previously reported in the literature [23]. 

Based on the fourth-order generalized Maxwell model and the introduction of BMD parameters, the constitutive equations of different BMD of CBAB were obtained by using the nonlinear least squares method to minimize the error, as shown in Equations (9) and (10):

(9) $$E^{\prime }=A\rho^{m}+A\left[\rho^{n}\sum_{i=1}^{n}\left(\frac{g_{i}\tau_{\mathrm{i}}^{2}\omega^{2}}{1+\tau_{i}^{2}\omega^{2}}\right)\right]$$

(10) $$E^{\prime \prime }=B\left[\rho^{q}\sum_{i=1}^{n}\left(\frac{g_{i}\tau_{i}\omega }{1+\tau_{i}^{2}\omega^{2}}\right)\right]$$

where *A*, *B*, *m*, *n*, and *q* are constants.

### 3.5. Fitting of Test and Models

Equations (9) and (10) were used to fit the experimental data of each sample, and the parameters obtained by fitting are shown in Table 3.

**Figure 8:**
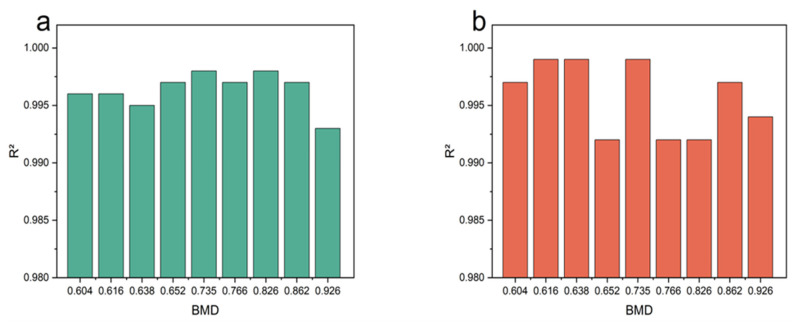
The fitting degree of model. (**a**) Storage modulus fitting test data. (**b**) Loss modulus fitting test data.

The determination coefficient R^2^ was used to evaluate the accuracy of the fitting. R^2^ varies between 0 and 1, so the closer the R^2^ value is to 1, the better the fitting is. The sample fitting curves of different BMD are shown in Figure 8, and it can be seen that the fitting was satisfactory (R^2^ > 0.991).

## 4. Discussion

Previous studies mostly used animal alveolar bone samples as substitutes and are mostly based on static tests. In fact, there is a significant difference between human and animal CBAB samples. In addition, human CBAB was also affected by dynamic chewing force during orthodontic treatment. Therefore, this study investigated the relationship between the microstructure of human CBAB and its mechanical properties by combining imaging and dynamic mechanics experiments. The samples used in this study were cut from the cervical, middle, and apical regions of the right mandible between canine, premolars, and molars. The BMD range of CBAB in different tooth regions was measured to be 0.604 g/cm^3^ to 0.926 g/cm^3^ based on micro-CT scanning. DMA described the properties of materials through the state of molecular motion [11]. Storage modulus was the energy storage capacity of viscoelastic materials under a cyclic alternating load. The larger the storage modulus is, the larger the stiffness of the material will be. Loss modulus represented the ability to dissipate energy within a period of changing and reflects the viscosity of the material. [16,24]. According to the results of DMA, when the frequency was increased by 10 times, and the average change rate of storage modulus and loss modulus were only about 25% and 28%. When the loading frequency was small, the macromolecular chains inside the material were still frozen, and the force was mainly transmitted through the movement of small molecular chains and segments [25,26]. With the increase in frequency, the cycle of alternating load was gradually smaller than the relaxation time of internal molecular chains and segments; the movement of segments cannot keep up with the change of stress, which was manifested as the increase in storage modulus and the enhancement of elastic properties. As the frequency increased, the oscillation force time acting on the CBAB became shorter. Then, the energy loss of the CBAB per unit time was weakened and the loss modulus was reduced. It is known that the ability of CBAB to dissipate energy (prevent stress concentration) decreases at higher loading frequencies (or impact loads). Moreover, it can be seen that the application of appropriate frequency force during orthodontic treatment is beneficial to the bone remodeling of CBAB. This was consistent with the conclusion of previous animal experiments that vibration can accelerate tooth movement [27]. However, when BMD increased by 53%, the average change rates of storage modulus and loss modulus were about 281% and 82%, respectively. The increase in BMD was the result of the increase in the number of trabecular bones with complex pores in CBAB (Figure 4) and the number and length of its internal molecular chains [11]. Therefore, the storage modulus was increased and the elastic properties were improved. Thus, for CBAB samples with larger BMDs, it was not easy to deform under cyclic loading. Through experiments and models, we learn that the alveolar bone has different bone mineral densities at different tooth positions. For alveolar cancellous bone with high bone mineral density, greater force is required to make it produce the same movement during orthodontic treatment. At the same time, the viscous substances such as bone marrow in CBAB also decreased accordingly, and the loss modulus also decreased. This means that the ability of CBAB to prevent stress concentration decreases, so a larger force should be applied to assist the treatment during orthodontic treatment, while avoiding stress concentration. At the same time, the viscous substances such as bone marrow in CBAB were reduced accordingly; thus, the loss modulus was reduced. It can be seen from the above that the effect of frequency on the overall change of viscoelastic modulus was much smaller than that of BMD. Therefore, this study focused on the effect of BMD on CBAB. We constructed a mechanical model of CBAB based on BMD distribution, which can help dentists evaluate orthodontic force in clinical treatment. For CBAB samples with larger BMDs, it was not easy for deformation under cyclic loading to occur, so larger forces should be applied to assist treatment during orthodontic treatment in these cases.

However, there are still a few limitations in the current experimental research. Due to the preciousness of the samples, all nine cancellous bone samples used in the study were from different parts of one human body, and biological repetition will be completed in the future with more data. On the other hand, the constructed model can evaluate the mechanical behavior of alveolar bone. During the orthodontic process, bone mineral density is in a long-term dynamic change state, and time can be considered a parameter that could optimize the model in the future.

## 5. Conclusions

In this paper, the experimental results of DMA showed that the BMD of human CBAB with different tooth regions has variability under dynamic loading, and BMD was one of the key factors affecting the mechanical properties of CBAB. The storage modulus and BMD were positively correlated, and the loss modulus and BMD were negatively correlated. Based on the fourth-order generalized Maxwell model, a viscoelastic constitutive model characterizing the change in BMD was constructed by using BMD as a parameter. The constructed model fitted well with the experimental data values (R^2^ > 0.991), which are able to accurately and efficiently describe the dynamic viscoelastic properties of CBAB. Evaluating the BMD of CBAB is useful for dental treatments, and this study not only provides a reference for the further study of the microstructure of CBAB but can also help dentists to assess orthodontic force in clinical treatment.

## Figures and Tables

**Table 1:** The BMD of the samples on different regions.

Tooth Regions	Bone Mineral Density (g/cm^3^)		
	Cervical Region	Middle Region	Apical Region
Region 3	0.826	0.638	0.604
Region 2	0.862	0.652	0.616
Region 1	0.926	0.766	0.735

**Table 2:** The number of bone trabeculae corresponding to bone mineral density.

Bone Mineral Density (g/cm^3^)	0.604	0.616	0.638	0.652	0.735	0.766	0.826	0.862	0.926
Trabecular number (1/pixel)	0.061	0.065	0.068	0.072	0.078	0.082	0.089	0.094	0.142

**Table 3:** The parameters of constitutive models.

*BMD*	*A*	*m*	*n*	*B*	*q*	*g_i_*				*τ_i_*			
						*g*_1_	*g*_2_	*g*_3_	*g*_4_	*τ*_1_	*τ*_2_	*τ*_3_	*τ*_4_
0.926	48.810	−38.381	−6.959	14.580	−7.91	0.999	0.972	0.581	0.996	0.022	0.431	0.103	3.844
0.766	17.970	−14.076	−4.305	15.373	6.701	0.405	0.383	0.828	0.735	0.126	0.045	0.533	0.012
0.735	3.291	−17.421	−8.118	3.368	−10.786	0.497	0.741	0.892	0.437	0.038	0.008	0.552	0.131
0.862	31.947	−22.076	−10.058	27.237	−3.148	0.999	0.839	0.500	0.023	0.522	0.023	0.111	0.003
0.652	12.497	−9.331	−0.762	12.082	−4.441	0.625	0.688	0.999	0.554	0.112	0.519	0.024	0.519
0.616	9.568	−7.677	−3.609	11.994	−4.790	0.584	0.890	0.578	0.997	0.042	0.010	0.145	0.584
0.826	22.326	−18.518	−3.935	45.121	−0.156	0.998	0.629	0.815	0.999	0.021	0.077	0.259	1.057
0.638	2.834	−11.381	−5.005	12.816	−4.790	0.824	0.959	0.527	0.520	0.011	0.581	0.142	0.044
0.604	9.604	−6.369	−3.774	12.081	−4.758	0.479	0.808	0.503	0.970	0.041	0.011	0.128	0.549

## Data Availability

Data are contained within the article.
